# Flowering and Runnering of Seasonal Strawberry under Different Photoperiods Are Affected by Intensity of Supplemental or Night-Interrupting Blue Light

**DOI:** 10.3390/plants13030375

**Published:** 2024-01-26

**Authors:** Jingli Yang, Jinnan Song, Byoung Ryong Jeong

**Affiliations:** 1 Shandong Provincial University Laboratory for Protected Horticulture, Weifang University of Science and Technology, Shouguang 262700, China or yangmiaomiaode@gmail.com (J.Y.); jinnansong93@gmail.com (J.S.); 2 Department of Horticulture, Division of Applied Life Science (BK21 Four), Graduate School, Gyeongsang National University, Jinju 52828, Republic of Korea; 3 Institute of Agriculture and Life Science, Gyeongsang National University, Jinju 52828, Republic of Korea; 4 Research Institute of Life Science, Gyeongsang National University, Jinju 52828, Republic of Korea

**Keywords:** carbohydrate accumulation, circadian rhythm, GA pathway, light intensity, photoperiodic response, photosynthetic efficiency, seasonal flowering, supplemental or night-interrupting blue light

## Abstract

The strawberry (*Fragaria × ananassa* Duch.) “Sulhyang” is a typical seasonal flowering (SF) strawberry that produces flower buds in day lengths shorter than a critical limit (variable, but often defined as <12 h). There is a trade-off between photoperiod-controlled flowering and gibberellin (GA) signaling pathway-mediated runnering. Some related genes (such as *CO*, *FT1*, *SOC1*, and *TFL1*) participating in light signaling and circadian rhythm in plants are altered under blue light (BL). Sugars for flowering and runnering are mainly produced by photosynthetic carbon assimilation. The intensity of light could affect photosynthesis, thereby regulating flowering and runnering. Here, we investigated the effect of the intensity of supplemental blue light (S-BL) or night-interrupting blue light (NI-BL) in photoperiodic flowering and runnering regulation by applying 4 h of S-BL or NI-BL with either 0, 10, 20, 30, or 40 μmol·m^−2^·s^−1^ photosynthetic photon flux density (PPFD) in a 10 h short-day (SD10) (SD10 + S-BL4 or + NI-BL4 (0, 10, 20, 30, or 40)) or 14 h long-day (LD14) conditions (LD14 + S-BL4 or + NI-BL4 (0, 10, 20, 30, or 40)). Approximately 45 days after the photoperiodic light treatment, generally, whether S-BL or NI-BL, BL (20) was the most promotive in runnering, leading to more runners in both the LD and SD conditions. For flowering, except the treatment LD14 + S-BL, BL (20) was still the key light, either from BL (20) or BL (40), promoting flowering, especially when BL acted as the night-interrupting light, regardless of the photoperiod. At the harvest stage, larger numbers of inflorescences and runners were observed in the LD14 + NI-BL4 treatment, and the most were observed in the LD14 + NI-BL (20). Moreover, the SD10 + NI-BL4 was slightly inferior to the LD14 + NI-BL4 in increasing the numbers of inflorescences and runners, but it caused earlier flowering. Additionally, the circadian rhythm expression of flowering-related genes was affected differently by the S-BL and NI-BL. After the application of BL in LD conditions, the expression of an LD-specific floral activator *FaFT1* was stimulated, while that of a flowering suppressor *FaTFL1* was inhibited, resetting the balance of expression between these two opposite flowering regulators. The SD runnering was caused by BL in non-runnering SD conditions associated with the stimulation of two key genes that regulate runner formation in the GA pathway, *FaGRAS32* and *FaGA20ox4*. In addition, the positive effects of BL on enhancing photosynthesis and carbohydrate production also provided an abundant energy supply for the flowering and runnering processes.

## 1. Introduction

The cultivated strawberry (*Fragaria × ananassa*) is an allo-octoploid that originated in the Americas more than 300 years ago. As a result of its sensory qualities and health benefits, strawberries have become one of the world’s most widely cultivated fruits [1,2,3,4,5]. The apical meristem of strawberries forms a determinate inflorescence on perennial rosette plants. In addition to bearing additional inflorescences or runners, their axillary meristems differentiate into branch crowns. The trade-off between flowering and runnering is a consequence of these alternate fates of axillary meristems [6,7]. There are two main groups of strawberries based on their flowering habits. A perennial flowering strawberry (PF) produces new floral inflorescences continuously once induced to flower, whereas a seasonal flowering strawberry (SF) produces flower buds under a critical day length limit (variable, but often defined as <12 h). In the current study, the garden strawberry (*Fragaria × ananassa* Duch.) “Sulhyang” is a typical SF strawberry.

*Fragaria vesca* (*F. vesca*), the diploid woodland strawberry that generated the octoploid cultivated strawberry [8], possesses two classical mutants that affect flowering and runnering. It has been demonstrated that PF and runnerless phenotypes are caused by residual mutations in the SF locus (SFL), as well as the runnering (R) locus [9]. The *F. vesca* homolog of *TERMINAL FLOWER1* (*FvTFL1*) has been identified independently by two groups as a possible candidate gene for SFL [10,11]. Koskela et al. [10] reported that *FvTFL1* exerts a strong floral repressor role that prevents seasonal flowering. A mutation was found in gene encoding of the GA20-oxidase (*FvGA20ox4*), which is the enzyme responsible for the biosynthesis of gibberellins (GAs). Axillary buds highly express this gene, and mutated enzymes cannot convert GA12 to GA20, the precursor of bioactive GA1 [12].

Researchers have found that at least part of the genetic pathway is conserved in woodland strawberries [10,13,14,15,16,17], based on studies of cultivated strawberries [13,14]. To regulate flowering, *FvTFL1* integrates environmental signals in SF genotypes, and flowers are only induced when this gene is downregulated at low temperatures below 13 °C or during short days (SDs) at 13–20 °C, which prevent flower induction by activating *FvTFL1* at high temperatures [16]. The photoperiodic regulation of *FvTFL1* is well understood, but the genes involved in temperature regulation remain unclear. *FLOWERING LOCUS T1* (*FvFT1*) is activated in leaves by woodland strawberry’s homolog of *CONSTANS* (*FvCO*), which results in upregulation of *SUPPRESSOR OF THE OVEREXPRESSION OF CONSTANS1* (*FvSOC1*) at the shoot apex during long days (LDs) [15,17]. A high level of *FvCO-FvFT1-FvSOC1* signaling leads to flowering in PF woodland strawberries without a functional *FvTFL1*, in contrast with SF genotypes where the pathway outcome is reversed by upregulation of *FvTFL1* by *FvSOC1* [15,17]. Despite the lack of understanding of flower induction, *FvFT3*, *APETALA1* (*FvAP1*), and *FRUITFULL* (*FvFUL*) genes that are activated at the shoot apex after SDs or cool temperatures downregulate *FvTFL1* should be explored further [10,13,14,18]. As a major activator of *FvFT1*, *FvCO* has been demonstrated to play a significant role in the leaves [19]. It has been shown, however, that the *Arabidopsis* external coincidence model is not directly applicable to woodland strawberries based on a comparison of *FvCO* and *Arabidopsis CO* diurnal expression rhythms under SD and LD conditions [20,21]. *FT* activation in the evening is accompanied by *CO* mRNA expression in *Arabidopsis* during the LD afternoon [22]. The *FvFT1* expression in the *tfl1* mutant Hawaiian-4 (H4) has a major peak in the evening and a secondary peak 4 h after dawn, whereas the *FvCO* expression is mostly at dawn [19]. Although *FvCO* is required by different mechanisms for both *FvFT1* expression peaks, this mechanism is unknown. *FvFT1* expression is also sensitive to the photoperiod in *FvCO* overexpression lines, suggesting light affects *FvCO* activity to some extent [19], possibly by stabilizing the protein, as in *Arabidopsis* [20].

Plant breeders and growers may be able to control the balance between vegetative and sexual reproduction by gaining a better understanding of the trade-off between flowering and runnering. A number of studies indicate that strawberry axillary meristems are controlled by GA. The runnerless woodland strawberry mutants were shown to grow runners after GA treatment by Guttridge and Thompson [23], and a recent mutant screen revealed a similar reversion, which led to the identification of the suppressor gene, *DELLA*, which encodes a GA signaling repressor [24]. In contrast, GA biosynthesis inhibitors increase strawberry yield and branching in cultivated plants [25,26]. It has also been shown that *FvSOC1* controls runner formation and regulates several GA biosynthetic genes, including *FvGA20ox4*, which encodes an enzyme that limits GA biosynthesis in the axillary buds [12,15]. According to these data, *FvSOC1* activates *FvGA20ox4* and possibly other GA biosynthesis genes in axillary buds, leading to high levels of bioactive GA1, SLR degradation, and runner development [1].

Additionally, higher *FvFT1* mRNA levels are associated with earlier flowering under a variety of environmental conditions, including varying light quality. *FvFT1*-dependent far-red light (FRL) at the end of the day promotes flowering, whereas red light has the opposite effect. Further, blue light (BL), which only promotes flowering weakly, suggests that phytochromes are the primary photoreceptors in controlling woodland strawberry flowering [27]. Similar to the SF strawberries, *Chrysanthemum morifolium* is also a kind of SD flowering plant. As chrysanthemum cultivation employs technical skills, the photoperiodic limitation on flowering time can be lifted by blackouts or artificial colored lighting, increasing the length of the day, or taking a night break to ensure consistently high-quality flowers. Supplemental light can appear in the form of valuable light added to regular light, or as additional light that extends the length of the day [28]. Night break (NB) interrupts the duration of darkness with lighting, creating modulated LD environments [29,30]. Chrysanthemum flowering is significantly regulated by BL, according to Higuchi et al. [31]. Plants grown under SD conditions under white light (WL) were inhibited from flowering effectively by monochromatic red light (RL), but less so by monochromatic blue light (BL) and fluorescent red light. All the plants flowered when supplied with 4 h of low BL in SD conditions, either supplementary or NI, and there were no significant differences between normal SD and low BL conditions [32]. Non-flowered plants, especially under LD conditions, were just delayed in flower bud formation by four hours of low-intensity supplemental blue light (S-BL) or night-interrupting blue light (NI-BL) treatment [32,33]. Moreover, our previous research has shown that both photosynthetic carbon assimilation and photoreceptor-mediated regulation influence SD plant chrysanthemum flowering under supplementing or night-interrupting BL [34]. When natural sunlight was extended during the first 11 h of the photograph period, either R or B light inhibited the growth of *Chrysanthemum morifolium* [35]. Thus, the flowering response to BL depends on cultivar-specific factors (such as intensity, photoperiod, supplementary, or NB).

Flowering is also affected by light intensity-related photosynthetic efficiency and carbon assimilation [36,37]. A low-light environment tends to delay plants’ first flowering, prolong their flowering period, and lower their flowering index. This is a phenomenon that has primarily been studied on tomatoes and some other flowering plants [38]. Plants growing under low light conditions accumulate less nutrients, and their photosynthesis is weaker than plants growing in a suitable light intensity environment. As a result, the floral buds develop later, the flowering nodes increase, and the quality of the buds decreases [39]. Additionally, under strong lighting, the effect of light intensity on flowering time is widely variable, with some studies showing that higher light intensities negatively affect flowering. Flowering time regulation may be related to changes in photosynthetic and carbon assimilation efficiency since light intensity affects photosynthesis and carbon assimilation efficiency. It has been suggested that phyA may regulate photosynthesis in *Arabidopsis* and that mutants of *phytochrome A* (*phyA*) show delayed flowering under low irradiation, but not high irradiation [40]. Moreover, the flowering of parthenogenic short-day plants was studied at different light intensities (42, 45, 92, and 119 mmol·m^−2^·s^−1^ PPFD), and it was found that the plants flowered earliest at low irradiance (42 mmol·m^−2^·s^−1^ PPFD) [41]. phyA may also regulate photoperiodic carbon assimilation, which modulates flowering regulation.

Here, a potted plant of the “Sulhyang” was used in this experiment to examine the photoperiod response of SD plant *Fragaria × ananassa* strawberry to different intensities of supplemental or night-interrupting blue light. Our results demonstrate that the flower–runner balance of seasonal strawberry in response to the intensity of supplemental or night-interrupting blue light is co-modulated by photosynthetic functionality and flowering–runnering-related genes.

## 2. Results

### 2.1. Morphology and Plant Growth Parameters

**Figure 1:**
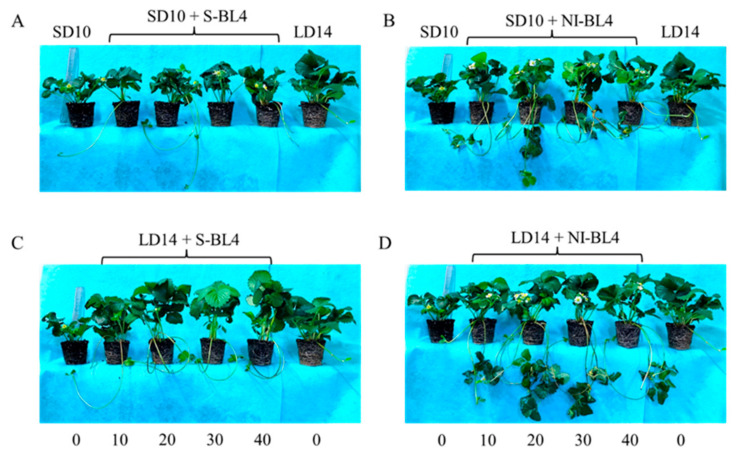
The morphology of strawberry “Sulhyang” under different intensities of supplemental or night-interrupting blue light for 45 days of exposure to the photoperiodic light treatments. Strawberry plants grown under short day 10 h conditions with 4 h of supplemental or night-interrupting blue light treatments (**A**,**B**); strawberry plants grown under long day 14 h conditions with 4 h of supplemental or night-interrupting blue light treatments (**C**,**D**). Numbers 0, 10, 20, 30, and 40 refer to the blue light intensity. See Figure 11 for details of photoperiodic light treatments with blue light.

**Figure 2:**
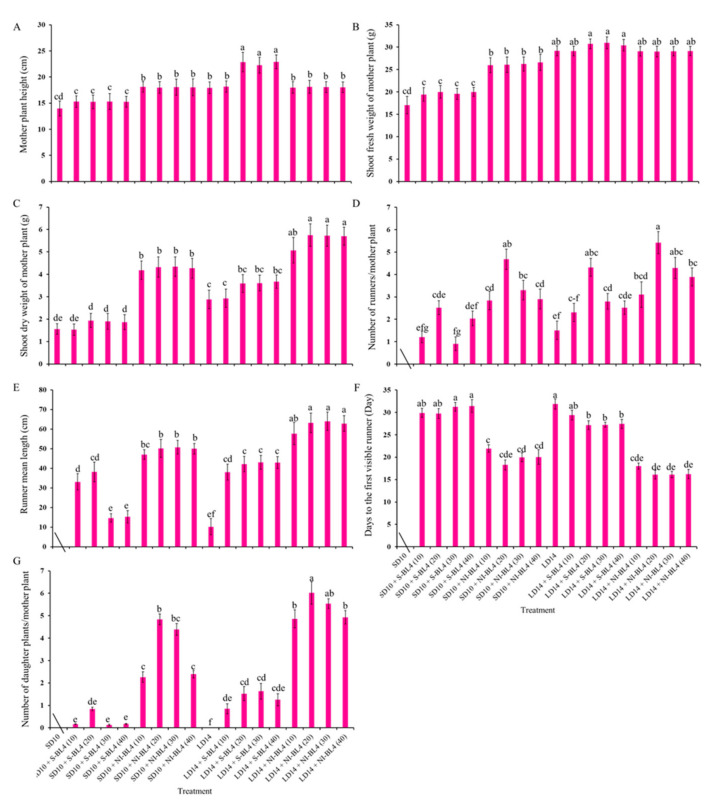
Runnering and growth parameters of strawberry “Sulhyang” under different intensities of supplemental or night-interrupting blue light for 45 days of exposure to the photoperiodic light treatments. Shoot height (**A**), fresh weight (**B**), dry weight (**C**), and average number of runners (**D**) and daughter plants (**G**) of mother plants; runner mean length (≥2 cm) (**E**) and days to the first visible runner (**F**). Strawberries in SD10 treatment were not included in the runner-related statistics. Vertical bars indicate the means ± standard error (*n* = 12). Different lowercase letters indicate significant separation within treatments by Duncan’s multiple range test at *p* ≤ 0.05. See Figure 11 for details of photoperiodic light treatments with blue light.

Figure 1 and Figure 2 present the plant morphological characteristics and growth parameters of the strawberry plants under different light treatments. In the current experiment, different light treatments had a significant effect on plant growth and development. In short-day conditions, the shoot height of the mother plants was considerably increased in the SD10 + NI-BL4 treatment when compared with those in the SD10 + S-BL4 and SD10 treatments. Mostly, the plant height showed there were non-significant differences under different light intensities in the same treatment. However, in long-day conditions, the LD14 + S-BL4 (20, 30, and 40) caused the highest plant height. Compared with the LD14 treatment, treatment LD14 + NI-BL4 had no effect on plant height (Figure 2A). Generally, the long day was more conducive to plant fresh weight accumulation than the short day. Specifically, the maximum shoot fresh weight of the mother plants was observed under LD14 + S-BL4 (20), (30), and (40), respectively (Figure 2B); however, the LD14 + NI-BL4 (20, 30, and 40) resulted in the maximum dry weight of the mother plants (Figure 2C). In this study, the strawberry plants in the SD10 treatment were not included in the runner-related statistics due to the runner inhibition in short day. However, blue light can promote the production of strawberry runners in a short-day environment, and the BL4 (20) performed particularly well, especially the SD10 + NI-BL4 (20), which caused the maximum number of runners and daughter plants per mother plant in short day, while LD14 + NI-BL4 (20) was always the best of all the light treatments in runner- or daughter plant-formation (Figure 2D,G). Except in SD10 + S-BL4, the BL4 (20, 30, and 40) resulted in the longest runner mean length compared to the other treatments; notably, the longest length was observed in treatment LD14 + NI-BL4 (20, 30, and 40) (Figure 2E). Moreover, treatment LD14 + NI-BL4 significantly reduced the days to the first visible runner formation, especially the LD14 + NI-BL4 (20, 30, and 40), which led to the earliest runner observation (Figure 2F). Whether using blue light as a supplement or night interruption, these results present that applying 4 h of blue light in both short-day and long-day conditions not only improves the traits related to the growth and development of strawberry plants, but also promotes their runner formation in varying degrees. In addition, the BL4 (20) acted as the key role in treatments SD10 + NI-BL4 and LD14 + NI-BL4 for runner formation.

**Figure 3:**
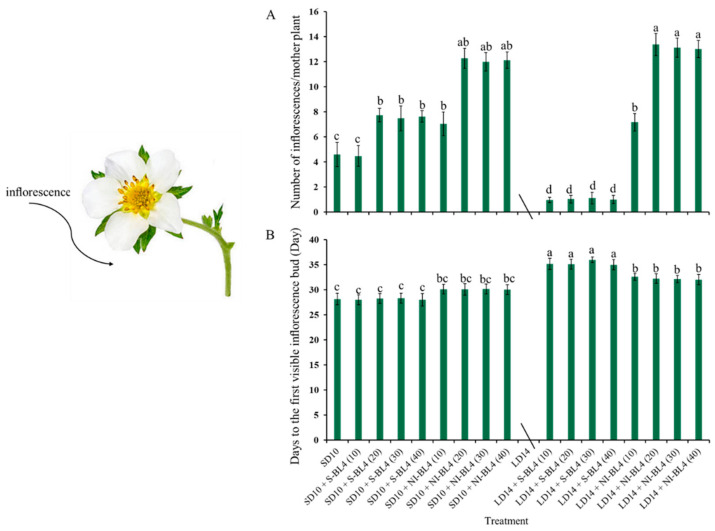
Flowering of strawberry “Sulhyang” under different intensities of supplemental or night-interrupting blue light for 45 days of exposure to the photoperiodic light treatments. Average number of inflorescences per mother plant (**A**) and days to the first visible inflorescences (**B**). Strawberries in LD14 treatment were not included in the statistics of inflorescences. Vertical bars indicate the means ± standard error (*n* = 12). Different lowercase letters indicate significant separation within treatments by Duncan’s multiple range test at *p* ≤ 0.05. See Figure 11 for details of photoperiodic light treatments with blue light.

Due to the photoperiodic flowering of strawberry *Fragaria ananassa* Duch. “Sulhyang” in long-day conditions, the strawberries in treatment LD14 were not included in the statistics of inflorescences (Figure 3). In short days, treatments SD10 + NI-BL4 (20, 30, and 40) caused more inflorescences than the SD10, SD10 + S-BL4 (10, 20, 30, and 40), and SD10 + NI-BL4 (10) treatments. In the long-day environment, the addition of blue light promoted the formation of inflorescence. In particular, treatment LD14 + NI-BL4 significantly increased the number of inflorescences per mother plant, and LD14 + NI-BL4 (20, 30, and 40) treatments formed the most inflorescences at the end of the experiment (Figure 3A). Compared with treatments SD10 and SD10 + S-BL4, SD10 + NI-BL4 slightly delayed the appearance of inflorescences in the strawberry plants, but the latest was in the long-day environment, especially treatment LD14 + S-BL4 (Figure 3B). The appearance of inflorescences was independent of the intensity of the blue light.

### 2.2. Photosynthetic Pigment Contents

**Figure 4:**
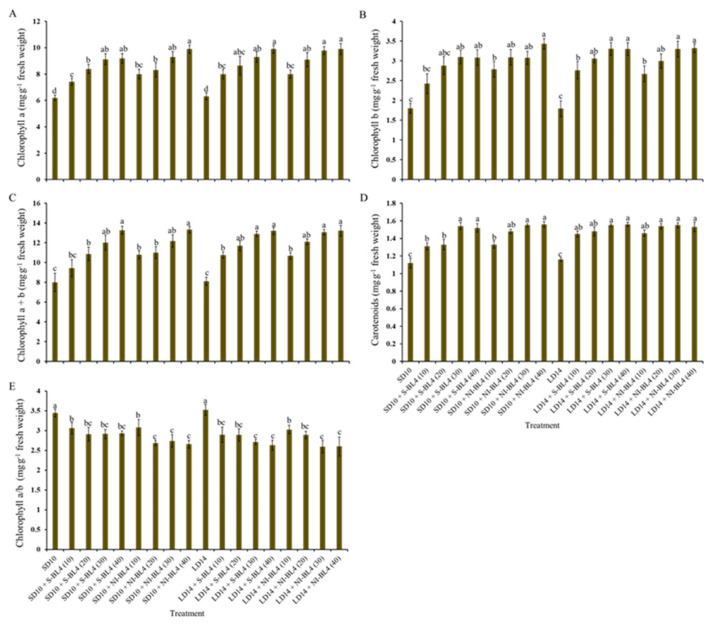
Photosynthetic pigment contents of strawberry “Sulhyang” under different intensities of supplemental or night-interrupting blue light for 45 days of exposure to the photoperiodic light treatments. Content of chlorophyll a (**A**), chlorophyll b (**B**), chlorophyll a + b (**C**), and carotenoids (**D**), and ratio of chlorophyll a to b (**E**). Vertical bars indicate the means ± standard error (*n* = 12). Different lowercase letters indicate significant separation within treatments by Duncan’s multiple range test at *p* ≤ 0.05. See Figure 11 for details of photoperiodic light treatments with blue light.

To further investigate how the effect of light intensity of supplemental or night-interrupting blue light under different photoperiods solely regulates the photosynthetic response in strawberry, the photosynthetic pigment contents (including chlorophylls a, b, a + b, and carotenoids) were determined in younger mature compound leaves of a strawberry mother plant at night. Whether it was S-BL4 or NI-BL4, the content variation trend of chlorophylls a, b, a + b, and carotenoids generally increased from BL4 (0) to BL4 (40), regardless of the photoperiod; generally, the maximum occurred in BL4 (30) and BL4 (40) (Figure 4A–D). In the opposite trend, the value of chlorophyll a/b gradually decreased from BL4(0) to BL4(40), and the largest chlorophyll a/b was observed in the SD10 and LD14 treatments (Figure 4E).

### 2.3. Photosynthetic Characteristics

**Figure 5:**
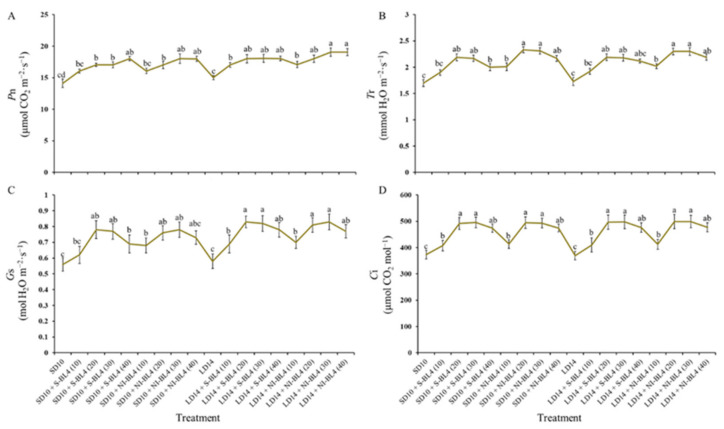
Photosynthetic characteristics of strawberry “Sulhyang” under different intensities of supplemental or night-interrupting blue light for 45 days of exposure to the photoperiodic light treatments. (**A**) Net photosynthetic rate (*P*n). (**B**) Transpiration rate (*T*r). (**C**) Stomatal conductance (*G*s). (**D**) Intercellular CO_2_ concentration (*C*i). Vertical bars indicate the means ± standard error (*n* = 12). Different lowercase letters indicate significant separation within treatments by Duncan’s multiple range test at *p* ≤ 0.05. See Figure 11 for details of photoperiodic light treatments with blue light.

Similar to the change trend of the photosynthetic pigment contents, for both S-BL4 and NI-BL4, the net photosynthetic rate (*P*n) increased gradually from BL4(0) to BL4(40) and reached its maximum value at BL4(30) or BL4(40), independent of the photoperiod (Figure 5A). Compared with *P*n, the changes of other photosynthetic traits (including transpiration rate (*T*r), stomatal conductance (*G*s), and intercellular CO_2_ concentration (*C*i)) were slightly different: their values gradually increased from BL4(0) to BL4(30) but decreased to almost the same level with BL4(0) at BL4(40) (Figure 5B).

### 2.4. Carbohydrate Contents

**Figure 6:**
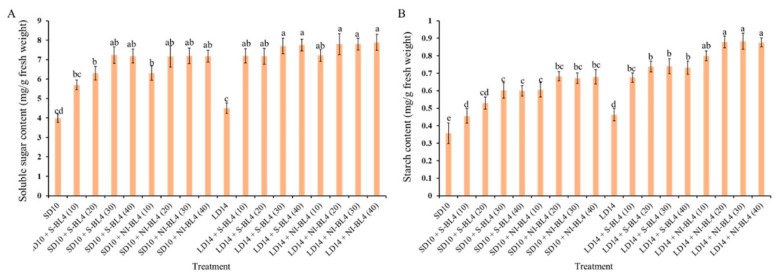
Carbohydrate contents of strawberry “Sulhyang” under different intensities of supplemental or night-interrupting blue light for 45 days of exposure to the photoperiodic light treatments. Soluble sugar content (**A**) and starch content (**B**). Vertical bars indicate the means ± standard error (*n* = 12). Different lowercase letters indicate significant separation within treatments by Duncan’s multiple range test at *p* ≤ 0.05. See Figure 11 for details of photoperiodic light treatments with blue light.

In the present experiment, the contents of starch and total soluble sugar were investigated under different treatments and showed various degrees of change trend. Regardless of S-BL4 or NI-BL4, the total soluble sugar content gradually increased from BL4 (0) to BL4 (40), reaching a maximum at BL4 (20), BL4 (30), or BL4 (40), independent of the photoperiod (Figure 6A). However, the starch content was generally higher in strawberries grown in the long-day environment than in the short-day environment. The starch content increased significantly from BL4 (10), but the change from BL4 (20) to BL4 (30) tended to be flat or there was no difference in either the S-BL4 or NI-BL4. The maximum starch content of the strawberry leaves was observed in treatments LD14 + NI-BL4 (20, 30, and 40) (Figure 6B).

### 2.5. Enzymatic Activities

**Figure 7:**
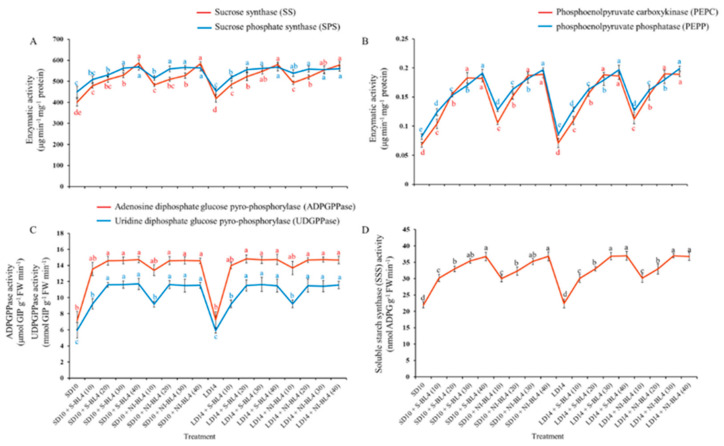
Enzymatic activities of strawberry “Sulhyang” under different intensities of supplemental or night-interrupting blue light for 45 days of exposure to the photoperiodic light treatments. Sucrose synthesis enzymes: (**A**) sucrose synthase (SS) and sucrose phosphate synthase (SPS), (**B**) phosphoenolpyruvate carboxykinase (PEPC) and phosphoenolpyruvate phosphatase (PEPP). Starch synthesis enzymes: (**C**) adenosine diphosphate glucose pyro-phosphorylase (ADPGPPase), uridine diphosphate glucose pyrophosphorylase (UDGPPase) and (**D**) soluble starch synthase (SSS). Vertical bars indicate the means ± standard error (*n* = 12). Different lowercase letters indicate significant separation within treatments by Duncan’s multiple range test at *p* ≤ 0.05. See Figure 11 for details of photoperiodic light treatments with blue light.

Different blue light intensity treatments resulted in significant differences in sucrose synthase (SS), sucrose phosphate synthase (SPS), phosphoenolpyruvate carboxykinase (PEPC), phosphoenolpyruvate phosphatase (PEPP), and soluble starch synthase (SSS). Regardless of the photoperiod, for both the S-BL4 and NI-BL4 treatments, the SS, SPS, PEPC, PEPP, and SSS activities of the strawberry plants gradually increased with increasing light intensity from BL4 (0) to BL4 (40), and higher values were measured under BL4 (30) or BL4 (40) than those in other light treatments (Figure 7A,B,D). The trend of activity expression of adenosine diphosphate glucose pyrophosphorylase (ADPGPPase) and uridine diphosphate glucose pyro-phosphorylase (UDPGPPase) was basically the same, with no change from BL4 (20) to BL4 (40), and they maintained the maximum value (Figure 7C).

### 2.6. Gene Expression

Because the short-day strawberry plant has a clear circadian rhythm, its flowering and runnering fluctuate a lot depending on the day and night conditions. Thus, the temporal expression patterns of blue light photoreceptors and flowering-related genes in the leaf or shoot apex of the strawberry mother plant under different intensities of supplemental or night-interrupting blue light for 10 days of exposure to the photoperiodic light treatments were investigated.

**Figure 8:**
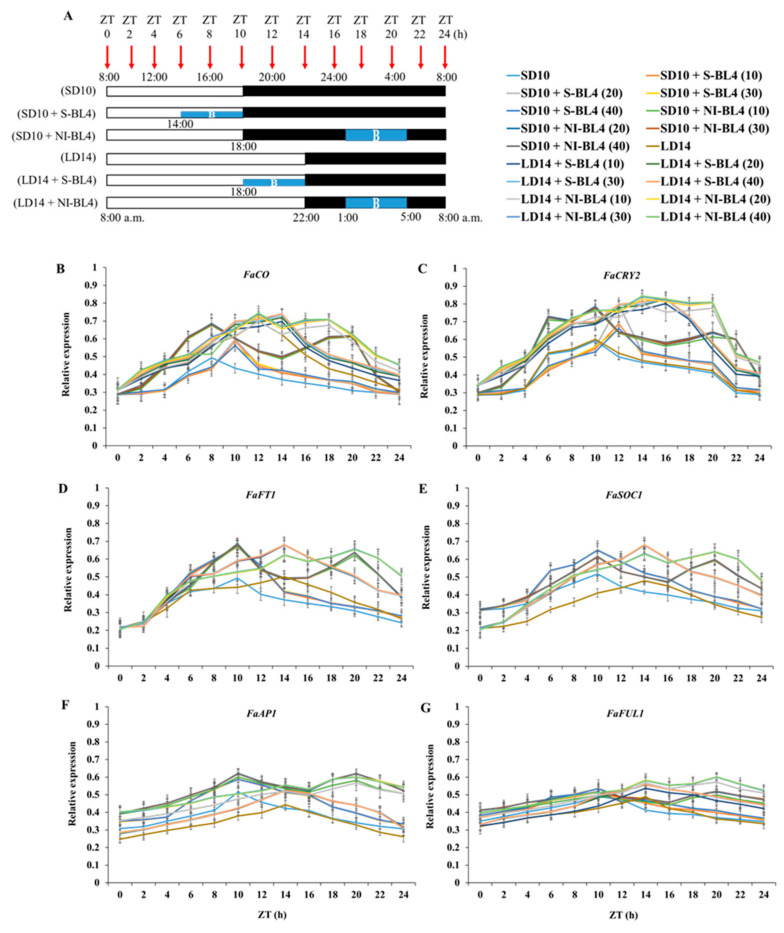
The temporal expression patterns of blue light photoreceptor or flowering-related genes in leaf or shoot apex of strawberry “Sulhyang” under different intensities of supplemental or night-interrupting blue light for 10 days of exposure to the photoperiodic light treatments. For RNA extraction and RT-PCR, the youngest mature compound leaves or new shoot apices were harvested at 0, 2, 4, 6, 8, 10, 12, 14, 16, 18, 20, 22, and 24h after lights-on (from 8:00 a.m.), respectively (ZT 0, 2, 4, 6, 8, 10, 12, 14, 16, 18, 20, 22, and 24). (**A**) The horizontal white and black bars represent the period of day and night, respectively; the blue bars represent the periods with different intensities of supplemental or night-interrupting blue light. (**B**) The *Fragaria ananassa* Duch. homologs of *CONSTANS* (*FaCO*), (**C**) *Cryptochrome2* (*FaCry2*), and (**D**) *FLOWERING LOCUS T1* (*FaFT1*) have been measured in the youngest ternately compound leaves. The *Fragaria ananassa* Duch. homologs of (**E**) *SUPPRESSOR OF THE OVER-EXPRESSION OF CONSTANS1* (*FaSOC1*), (**F**) *APETALA1* (*FaAP1*), and (**G**) *FRUITFULL1* (*FaFUL1*) have been measured in the shoot apex samples. Data were averagely normalized against the expression of *FaEF1* (Acttin gene-1) and *FaMSI1* (Acttin gene-2). The maximum value in each experiment was set to “1”. Vertical bars indicate the means ± standard error (*n* = 12), using RNA from separate plants. See Figure 11 for details of photoperiodic light treatments with blue light. See Table 1 for the detailed primer information.

The overall expression of *FaCO* was accumulated in the daytime and degraded in the dark. In the SD10 treatment, the highest single peak appeared 8 h after the light was turned on. In the SD10 + S-BL4 treatment, the highest peak value was reached at 18:00, when both the blue and white lights were turned off, and then decreased, with no significant difference between the different treatments. In the SD10 + NI-BL4 treatment, the first peak was reached at 16:00, and the second peak was reached 20 h after the light was turned on. The first peak was slightly higher than the second peak, and there was no significant difference between the intensity. In the LD14 treatment, the highest peak appeared at 20:00 and then decreased gently, and the peak value in the LD14 treatment was higher than the single peak value in the SD10 treatment. In the LD14 + S-BL4 treatment, the highest peak appeared at 22:00 and then decreased, and the peak of BL4 (10) was lower than the peak of the other three light intensities. In LD14 + NI-BL4 processing, there was a double high peak, which was higher than the single-peak height. The first peak occurred 12 h after the light was turned on, and the second peak occurred 18 h after the light was turned on. The first peak was slightly higher than the second peak, and the peak of BL4 (10) was lower than the peak of the other three light intensities (Figure 8B).

In the SD10 treatment, *FaCRY2* expression increased sharply 4 to 6 h after the light was turned on, reached a maximum value at 18:00, and then plummeted 20 to 22 h after the light was turned on. In the SD10 + S-BL4 treatment, the highest peak value was reached 12 h after the light was turned on, and the peak value was the largest in BL4 (20), followed by BL4 (10); there was no significant difference between BL4 (30) and (40). In the SD10 + NI-BL4 treatment, double peaks appeared, reaching the first peak and the second peak at 10 and 20 h after the light was turned on, respectively, and there was no significant difference between the different intensities. *FaCRY2* expression in LD14 showed the same trend as that in the SD10 treatment, with a sharp increase 4 to 6 h after the light was turned on, reaching a maximum value at 18:00, and then a sharp decline 20 to 22 h after the light was turned on. In the LD14 + S-BL4 treatment, the peak value was reached 16 h after the light was turned on, and the peak value at BL4 (10) was significantly lower than that at BL4 (20), (30), and (40). In the LD14 + NI-BL4 treatment, double peaks appeared at 14 and 20 h after the light was turned on, respectively, and the peaks at BL4 (10) were significantly lower than those of the other three light intensities (Figure 8C).

For *FaFT1* expression, in treatment SD10, the peak was reached 10 h after the lights were turned on. The trend of the SD10 + S-BL4 treatment was basically the same as that of SD10, but the peak value was significantly higher than SD10, and there was no significant difference between different light intensities. In the SD10 + NI-BL4 treatment, double peaks appeared at 10 and 20 h after the light was turned on, and there was no significant difference between different light intensities. A single peak value appeared in both LD14 and LD14 + S-BL4 and reached the maximum value 14 h after the light was turned on, but the peak value in LD14 + S-BL4 was significantly higher than that of LD14. Moreover, there was no significant difference between the different light intensities. In the treatment of LD14 + NI-BL4, the double peak values were reached at 14 h and 20 h after the light was turned on, and the secondary peak value was the largest, but there was no significant difference between different light intensities (Figure 8D).

The initial expression of *FaSOC1* in the short-day environment was significantly higher than that in the long-day environment. Regardless of the light intensity, the SD10 and SD10 + S-BL4 treatments peaked 10 h after the light was turned on, and the peak amplitude increased significantly after the addition of blue light. After the light was turned on for 14 h, the peak values of the LD14 and LD14 + S-BL4 treatments appeared simultaneously, and the peak value of LD14 was significantly smaller than that of the LD14 + S-BL4 treatment. In the LD14 + NI-BL4 treatment, the first peak also appeared at 14 h after the light was turned on, but the second peak appeared at 20 h after the light was turned on. The light intensity has no effect on the peak value (Figure 8E).

As shown in Figure 8F, for *FaAP1* expression, in the treatments of SD10 and SD10 + S-BL4, the peak value was reached 10 h after the light was turned on, and the blue light supplementation significantly increased the peak value, but the peak value did not respond to the light intensity. In the SD10 + NI-BL4 treatment, except for the first peak 10 h after the light was turned on, there was a second peak 20 h after the light was turned on, and the peak value of BL4 (10) was significantly smaller than that of BL4 (20, 30, and 40). In the LD14 and LD14 + S-BL4 treatments, the peak value was reached 14 h after the light was turned on, and the blue light was conducive to the increase in the peak value. There was no significant difference between the different light intensities. In the LD14 + NI-BL4 treatment, the first peak and the second peak were reached after 14 and 20 h, respectively, and the second peak was significantly higher than the first peak. Moreover, the peak value of BL4 (10) was significantly smaller than that of BL4 (20, 30, and 40). The expression trend of *FaFUL1* and *FaAP1* was basically the same, but the fluctuation of the expression value was small, which shows that the trend line was flatter (Figure 8G).

**Table 1:** PCR conditions and primers used to quantify gene expression.

Name	Forward Primer (5′ to 3′)	Reverse Primer (5′ to 3′)
*FaEF1* (Acttin gene-1)	TGGATTGCGATCCTCCAAAGT	AGAGCTTGTTGGCTGAAAGGA
*FaMSI1* (Acttin gene-2)	TCCCCACACCTTTGATTGCCA	ACACCATCAGTCTCCTGCCAAG
*FaCO*	GACATCCACTCCGCCAAC	GTGGACCCCACCACTATCTG
*FaCRY2*	CCATGGGATGTTAATGATG	CACCTGATATGATTCTCTTAG
*FaFT1*	CAATCTCTTGGCCGAAAACT	TGAGCTCAAACCTTCCCAAG
*FaSOC1*	ACATACAAGAGACAACCAAGCCA	GTTCCTTGAAAACCTGTGCCT
*FaTFL1*	AACGGCAGCAACAGGAAC	CTGGCACCACAGATGCTACA
*FaAP1*	AGCTCAGGAGGTTCATGACTG	TAAGGTCGAGCTGGTTCCTC
*FaFUL1*	GCAGTGCATGAATCCCTTTC	GCTGGTGATTTTGGAGCTTG
*FaRGA1*	GGGCTGTCTCATCTGGCTTC	GCAGTCCTCAAACTGGGTTC
*FaGRAS32* (*LAM*)	TCCGCTCACGGCCTTATTTC	AAGCGTCTCTTCTTCCGCTT
*FaGA20ox4*	AGCTCATTAGGGCTTCTTGCT	TCCATTCTCATGGAAGCCGAA
PCR conditions	PCR was performed with an initial denaturing step at 95 °C for 5 min, followed by 40 cycles at 95 °C for 5 s, 60 °C for 20 s, 72 °C for 30 s, and 72 °C for 10 min to final extension. Fluorescence was quantified after the incubation at 72 °C.	

**Figure 9:**
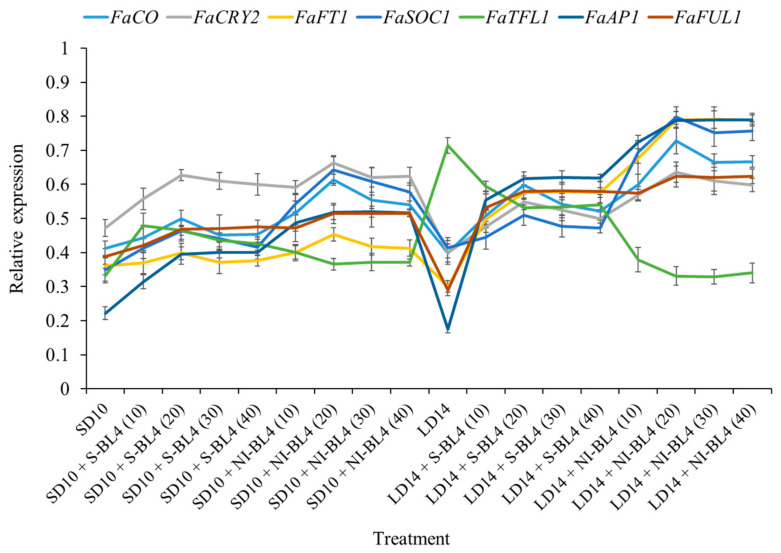
Expression patterns of flowering-related genes in the youngest ternately compound leaf or new shoot apex of strawberry “Sulhyang” under different intensities of supplemental or night-interrupting blue light for 45 days of exposure to the photoperiodic light treatments. The youngest ternately compound leaves or new shoot apices were harvested at ZT4 (4 h after lights-on, from 8:00 a.m.) for RNA extraction and RT-PCR. The *Fragaria ananassa* Duch. homologs of *CONSTANS* (*FaCO*), *Cryptochrome2* (*FaCry2*), and *FLOWERING LOCUS T1* (*FaFT1*) were measured in the youngest mature compound leaves. The *Fragaria ananassa* Duch. homologs of *SUPPRESSOR OF THE OVER-EXPRESSION OF CONSTANS1* (*FaSOC1*), *APETALA1* (*FaAP1*), *FRUITFULL1* (*FaFUL1*), and *TERMINAL FLOWER1* (*FaTFL1*) were measured in the shoot apex samples. Data were averagely normalized against the expression of *FaEF1* (Acttin gene-1) and *FaMSI1* (Acttin gene-2). The maximum value in each experiment was set to “1”. Vertical bars indicate the means ± standard error (*n* = 12) using RNA from separate plants. See Figure 11 for details of photoperiodic light treatments with blue light. See Table 1 for the detailed primer information.

The expression patterns of the flowering-related genes in the youngest ternately compound leaf or new shoot apex of the strawberry “Sulhyang” under different intensities of supplemental or night-interrupting blue light for 45 days of exposure to the photoperiodic light treatments were also investigated (Figure 9). Their expression patterns can be broadly classified into two types: (1) the anti-florigenic gene *FaTFL1* was expressed in this way and was significantly higher in the LD14 treatment, followed by LD14 + S-BL4, which observably inhibited flowering or resulted in no flowers. However, its expression decreased sharply in the LD14 + NI-BL4 treatment, which was basically equal to or even lower than that in all the short-day conditions. (2) The expression patterns of the other florigen genes, such as *FaCO*, *FaCRY2*, *FaFT1*, *FaSOC1*, *FaAP1*, and *FaFUL1*, were roughly opposite to *FaTFL1*, reaching the lowest value in the LD14 treatment and generally high expression in the LD14 + NI-BL4 treatment, especially at BL4 (20). The general rule of these floral genes is that they are proportional to the flowering capacity. Overall, these expression levels were correlated with the extent of flower induction in this study.

**Figure 10:**
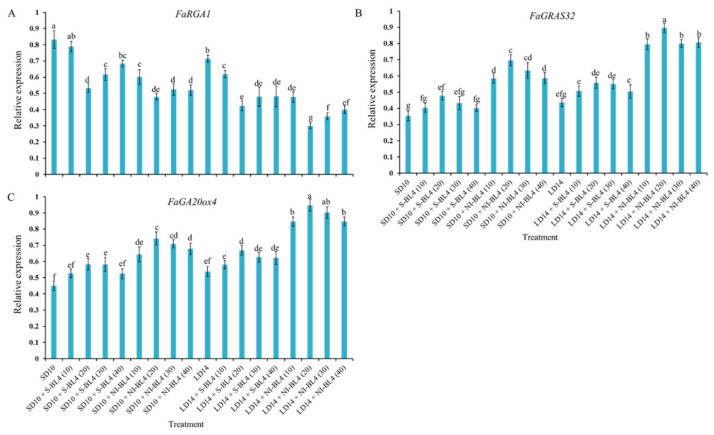
Expression patterns of runnering-related genes in the youngest ternately compound leaf or new shoot apex of strawberry “Sulhyang” under different intensities of supplemental or night-interrupting blue light for 45 days of exposure to the photoperiodic light treatments. The youngest ternately compound leaves or new shoot apices were harvested at ZT4 (4 h after lights-on, from 8:00 a.m.) for RNA extraction and RT-PCR. The *Fragaria ananassa* Duch. homologs of (**A**) the *REPRESSOR OF GA1* (*FaRGA1*) and (**B**) *GRAS transcription factor family* (*FaGRAS32*) were measured in the shoot apex samples. The *Fragaria ananassa* Duch. homolog of (**C**) the gibberellic acid (GA) biosynthesis gene *Gibberellin 20-oxidase 4* (*FaGA20ox4*) was measured in the youngest ternately compound leaves. Data were averagely normalized against the expression of *FaEF1* (Acttin gene-1) and *FaMSI1* (Acttin gene-2). The maximum value in each experiment was set to “1”. Vertical bars indicate the means ± standard error (*n* = 12) using RNA from separate plants. Different lowercase letters indicate significant separation within treatments by Duncan’s multiple range test at *p* ≤ 0.05. See Figure 11 for details of photoperiodic light treatments with blue light. See Table 1 for the detailed primer information.

To study the tissue-specific expression patterns of the runnering-related genes in *Fragaria ananassa* Duch., the runnering repressor gene *FaRGA1* and the runnering-promoted genes *FaGRAS32* and *FaGA20ox4* were selected and analyzed by qRTPCR in the shoot apexes and leaves, respectively (Figure 10). After 45 days of exposure to the photoperiodic light treatments, at the harvest stage, these runner-forming-related genes were all highly expressed in the shoot apices or leaves. The expression of the runnering repressor gene FaRGA1 was generally higher in the SD than in the LD conditions, especially in the SD10 and SD + S-BL4 (10) treatments, which significantly inhibited runnering or prevented a runner. The general rule of this runnering repressor gene is inversely proportional to the runnering capacity. The expression patterns of the other two runner-forming-promoted genes, *FaGRAS32* and *FaGA20ox4*, were roughly the opposite of *FaRGA1* and were highly expressed in the LD14 + NI-BL4 treatment, especially in BL4 (20). The same pattern was also reflected in the SD10 + NI-BL4 treatment, and the expression levels of these runner-forming-promoted genes were more responsive to BL4 (20) than the other light intensities.

## 3. Discussion

### 3.1. Strawberry Growth and Physiological Traits in Response to Various Intensities of Supplemental or Night-Interrupting Blue Light in Photoperiodic Treatments

A plant’s growth and development are affected by lights of different durations (photoperiods), intensities, and qualities [42]. Higher daily light intensity increases the strength of growth and yield of integral plants within an appropriate range of lighting intensity; hence, optimal light intensities and longer photoperiods are generally better for enhanced vegetative growth [43]. In SD periods, plants tend to limit their growth in order to save accumulated sugars because the non-produced nighttime lasts longer than the light time [44]. In the current study, all the LD conditions improved the shoot fresh weight and plant height compared to the SD conditions (Figure 1 and Figure 2A,B). It has been found that SDPs grow only nutritionally in LD conditions and do not flower there [45]. As we observed, LD14 caused SDP chrysanthemum to grow more vegetatively with no flowering (Figure 1, Figure 2 and Figure 3).

As well as photoreceptors and signal transduction, pigment biosynthesis, carbon metabolism, and nitrogen metabolism, there are various aspects related to the BL effect in plants. The BL environment is usually associated with shorter plants, thicker stems, and higher protein and carbohydrate contents [46]. As indicated by our results (Figure 4 and Figure 5), BL is essential in plants in order to produce chlorophyll and chloroplasts and to build high photosynthetic rates [47]. The PSII reaction center complex core protein D1 (QB) is synthesized de novo by the BL [48], thereby promoting photosynthesis (Figure 5). Blue light (BL)-grown plants have higher stomatal conductance, transcript levels of key photosynthetic genes, total soluble sugars, and starch content than white light (WL)-grown plants [49].

Moreover, the BL not only activates many enzymes in the carbohydrate synthesis, photosynthetic carbon assimilation and photorespiration, and chlorophyll synthesis pathways, but also induces the uridine diphosphate glucose (UDPG), pyrophosphorylase, and PEPC [50] to enhance nicotinamide adenine dinucleotide phosphate (NADP)-dependent phosphoglycerate dehydrogenase [51], which supports the results in Figure 7. A significant improvement was observed for S-BL4 and NI-BL4 under photoperiodic light treatment, supported by their positive effects on plant growth and development. The morphological and physiological traits, which are discussed above, were more improved after the photoperiodic light treatment. Regardless of the photoperiod, the 20, 30, and 40 mmol m^−2^ s^−1^ PPFD of S-BL4 or NI-BL4 improved strawberry growth and development more efficiently. Overall, the growth and physiology of strawberries is promoted by various intensities of S-BL4 or NI-BL4 in photoperiodic light treatments. However, the BL does not regulate plant development completely alone, but rather interacts with the intensity and photoperiod.

### 3.2. Strawberry Flowering in Response to Various Intensities of Supplemental or Night-Interrupting Blue Light in Photoperiodic Treatments

As shown in this study, the application of blue light, especially the SD10 + NI-BL4 treatment, could significantly increase flower formation under short-day conditions. In particular, this SD flowering strawberry, after BL treatment, actually formed flowers in an LD environment, and LD14 + NI-BL4 produced the most flowers of all the treatments. In different photoperiods, whether BL is used as supplementary light or interrupting light at night, the behavior of BL promoting flowers is significantly increased from BL4 (20) (Figure 3A). Flowering is highly dependent on the R/B light ratio [52]. In an LED system, BL led to increased flowering of woodland strawberries [27]. Using LED as a light source, Ye et al. (2021) exposed strawberry seedlings to BL or WL. When the cultivated strawberries were treated with BL instead of WL, flowering was significantly increased [53]. Researchers have reported similar findings on woodland strawberries and petunias [54].

Plants’ circadian clocks enable them to respond optimally to external environmental conditions by interpreting different wavelengths and intensities of light, as well as photoperiodic durations [55]. Figure 8 shows the time fluctuation expression of flowering-related genes, and BL interferes with their expression trend. In general, BL supplementation tends to form a single peak, while night-interrupting light generally forms a double peak, and BL contributes to the expression of flowering positive genes. A single repressor gene called *SEASONAL FLOWERING LOCUS* (*SFL*) has been shown to cause perpetual flowering by classic genetic studies [9,56]. Strawberries are shown to respond to photoperiodically controlled flowering by activating and inhibiting signals [57,58,59]. Koskela et al. (2012) confirmed that both signals are present in *F. vesca* and that SFL is a major switch controlling photoperiodic responses, and provided functional evidence that *SFL* encodes a *Fragaria* homolog of the floral repressor TFL1 and demonstrates that *FvTFL1* is photoperiodically regulated. They suggested that in the SD accessions of *F. vesca*, down-regulation of *FvTFL1* under SD allows flower induction to occur only in the autumn, which leads to seasonal flowering the next spring. In contrast, a mutation in *FvTFL1* causes rapid FT-dependent LD flowering and continuous initiation of inflorescences in the perpetual flowering LD accessions [10]. As the current results show, the expression of the flowering suppressor gene *FaTFL1* was particularly high in the LD condition without blue light, which is one of the reasons for non-flowering under the LD14 treatment (Figure 9).

Koskela et al. (2012) proposed that *FvTFL1* overcomes the function of the LD-specific floral activator *FvFT1* [10]. Both annuals and perennials are controlled by CO/FT modules [22,60,61], while FT is believed to be a universal signal for flowering [62,63,64]. As shown in Figure 9, the *FaFT1* expression level was particularly low in the non-flowering LD14 treatment, but BL4 (10) began to increase significantly in the LD14 + NI-BL4 treatment and reached its maximum expression level at LD14 + NI-BL4 (20). Its expression trend was completely opposite of that of the flowering inhibitory factor *FaTFL1.* Koskela et al. (2012) observed that the expression of *FvFT1*, a likely ortholog of *FT*, correlated with flowering in LD accession Hawaii-4. Furthermore, *FvFT1* RNAi plants exhibited a strong reduction in *FvAP1/FUL* gene expression and a late flowering phenotype, suggesting that *FvFT1* regulates flowering in *F. vesca* LD accessions [10]. Koskela et al. (2012) suggested that in this genotype, *FvTFL1* expression at the shoot apex may overcome *FvFT1*’s function as a flowering activator. Different external loops cause FT and TFL1 to function oppositely [10,65]. Together with FD and 14-3-3 proteins, FT forms a “florigen activation complex” [66]. *FvTFL1* may inhibit flowering by competing with *FvFT1* for binding partners because TFL1 homologs can also bind FD and 14-3-3 [67,68,69]. The balance between the expression of *FT* and *TFL1* homologs controls flowering in tomatoes (*Solanum lycopersicum*) [70]. However, in SD *F. vesca*, both *FvFT1* and *FvTFL1* are downregulated under flower-inductive conditions. Therefore, they hypothesize that flower induction in SD *F. vesca* takes place via an *FvFT1*-independent mechanism, whereas in Hawaii-4, *FvFT1* functions as an LD-specific floral promoter in the absence of functional *FvTFL1*. However, it is possible that FT is involved in the LD activation of *FvTFL1*, which is photoperiodically regulated only at the shoot apex [10]. *FvTFL1* photoperiodic control may require a systemic signal since photoperiod perception occurs in the leaves [71,72]. As a general photoperiodic signaling molecule, FT is a good candidate for such a signal [64]. Further, Hecht et al. (2011) demonstrated that leaf-expressed *FT* controls photoperiodic expression of another *CETS* family gene at the shoot apex of pea (*Pisum sativum*). After the downregulation of *FvTFL1*, further studies are needed to determine how floral meristem identity genes are activated in SD *F. vesca* [73].

### 3.3. Strawberry Runnering in Response to Various Intensities of Supplemental or Night-Interrupting Blue Light in Photoperiodic Treatments

Runners play an important role in strawberry economics since strawberries are clonally propagated. Plants have runners at their first internode at the leaf axil, which are elongated axillary buds. The plant hormone GA induces runnering [23,26,58,74]. If GA is produced or supplied, axillary buds take on a runner identity for outgrowth. Feng et al. (2021) suggested that GA plays a more significant role in strawberry runner outgrowth than in bud initiation [75]. In a wide range of plant species, GA represses bud initiation [76,77,78,79,80]. To determine whether GA plays a role in bud initiation, further experiments, such as over-expression of the *GA20ox* or *GA2ox* genes in strawberries, might be conducted.

A GA biosynthesis gene (*FveGA20ox4*) and a GA signaling gene (the *DELLA* gene *FveRGA1*) have been identified as key regulators of runner formation (mainly outgrowth) [12,24,81]. *FvSOC1* also induces GA biosynthesis, which is required for runner formation [15]. LAM (RGA1) and the GA pathway are sequentially involved in bud initiation and outgrowth, according to Feng et al. (2021) [75]. In this study, the expression of the runnering repressor gene *FaRGA1* was generally higher in the SD than in the LD conditions, especially in the SD10 and SD + S-BL4 (10) treatments, which significantly inhibited runnering. The general rule of this runnering repressor gene was inversely proportional to the runnering capacity (Figure 10). The LAM homolog MOC1 promotes rice tiller bud growth [82]. Identifying the exact role played by LAM in regulating runner outgrowth in strawberries would be possible by studying gain-of-function mutants of the protein. As with the tiller in rice, strawberries have an axillary meristem that forms the branch crown. MOC1 and SLENDER1 (SLR1) positively regulate tillering in rice, since the GA pathway negatively regulates it [77,82,83]. Similarly, the *FveRGA1* homolog SLR1 inhibits MOC1 degradation in rice by physically interacting with MOC1 [83]. Unlike rice MOC1 and SLR1, *lam* has fewer branch crowns than WT, a phenotype similar to *srl*. This suggests that LAM and *FveRGA1* might be involved in branch crown development in a similar manner. These protein–protein interactions may also occur in meristematic tissues because of the overlapping expression patterns of LAM and FveRGA1 [24].

It has been shown that *CO*, *SOC1*, *FveGA20ox4*, and *DELLA* are involved in stolon formation [12,15,17,24,84]. GRAS family proteins, such as DELLA, repress GA signaling through a number of mechanisms during growth and development [85]. Moreover, *FveGRAS34* contains a full DELLA motif, and a runnerless variety can be revived by the mutation of *FveGRAS34* [24]. The inhibition of *FveRGA1* expression (DELLA, gene06210) in naturally non-runnering woodland strawberry cultivars “Ruegen” and “Yellow Wonder” produced many runners [84], demonstrating that this DELLA protein controls the formation of runners during woodland strawberry asexual reproduction [24,84,85]. We found that *FaGRAS32* was highly expressed in the LD14 + NI-BL4 (10, 20, 30, and 40) treatments (Figure 10), which suggests that *FaGRAS32* participates in biological processes related to GAs in woodland strawberry. In order for axillary buds to develop into branch crowns or stolons, the photoperiod and temperature must be sensed by the leaves, so there may be a signaling pathway from the leaf to the crown that regulates the stolon or branch crown development [15,86,87]. There is a possibility that *FveGRAS* genes expressed in the crown, stolon, stolon tip, leaf, or petiole might control forest strawberry stolon and branch crown initiation or elongation; however, more research is needed. In particular, the stimulation of blue light on runnering-promoted gene expression and the most effective blue light intensity analysis remain to be further explored.

## 4. Materials and Methods

### 4.1. Plant Materials and Growth Conditions

Runners of the cultivated strawberry (*Fragaria* × *ananassa* Duch.) “Sulhyang” were obtained from a commercial strawberry farm (Sugok-myeon, Jinju, Gyeongsangnam-do, Republic of Korea) in mid-September of 2022, which shows seasonal short-day (SD) flowering. Before the light treatments started, runners with 3 ± 1 leaves per plant were raised in a greenhouse. Fluorescent lamps (F48T12-CW-VHO, Philips Co., Ltd., Eindhoven, The Netherlands) were used to supplement natural light with an average light intensity of 270 ± 5 μmol·m^−2^·s^−1^ PPFD. All the runners were kept on a fogged propagation bench with 80% relative humidity for 15 days. After rooting, the rooted runners were transplanted to 10 × 10 cm plastic pots (Daeseung, Jeonju, Republic of Korea) for the subsequent light treatments. A commercial medium (BVB Medium, Bas Van Buuren Substrates, EN-12580, De Lier, The Netherlands) supplemented with 25% (*v*/*v*) of vermiculite (Ø2 mm) was used as a growing media. The plants were fertilized with liquid fertilizer (macro-elements: Ca^2+^, Mg^2+^, K^+^, NH_4_^+^, NO_3_^−^, SO_4_^2−^, and H_2_PO_4_^−^; microelements: B, Cu, Fe, Mn, Mo, and Zn; pH = 6.5) weekly [88].

### 4.2. Light Treatments

A closed-type plant factory (770.0 cm long by 250.0 cm wide by 269.5 cm high, Green Industry Co. Ltd., Changwon, Republic of Korea) was used for the light treatments at 20 °C and 70 ± 5% relative humidity (RH). An electrolyte CO_2_ sensor (Model No. GMT220 Carbocap, Vaisala, Vantaa, Finland) monitored online the CO_2_ concentration of 350 ± 50 parts per million (PPM) from a compressed gas tank to supplement plant photosynthesis. Air circulated in the development rooms horizontally through multiple apertures that were evenly distributed throughout the system.

**Figure 11:**
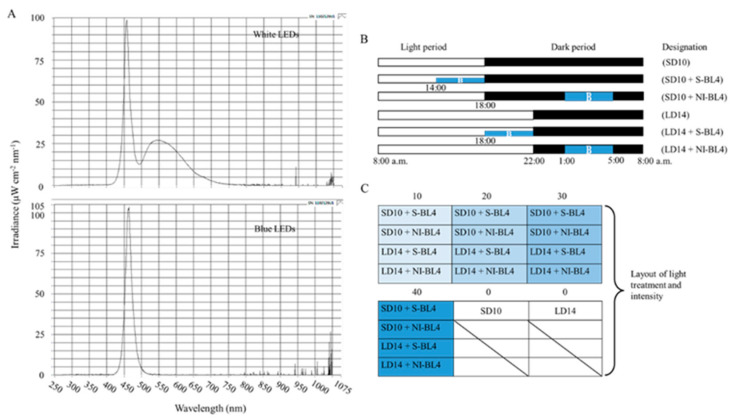
The light spectral distribution of experimental light treatments (**A**): the white light (~400–720 nm and peaked at 450 nm) provided by white LEDs and blue light (peaked at 450 nm) from blue LEDs used as the supplemental or night-interrupting light. The experimental light schemes employed in this study (**B**): the light period (the white bar) started and the dark period (the black bar) ended at everyday 8:00 a.m.; plants in the control groups were in a 10 h short-day (SD10) or 14 h long-day (LD14) condition, without any blue light; the 4 h blue light (the blue bar) with either 10, 20, 30, or 40 μmol·m^−2^·s^−1^ PPFD of intensities was used to (1) supplement the white light at the end of the SD10 (SD10 + S-BL4) and LD14 (LD14 + S-BL4) or (2) provide night interruption (NI) in the SD10 (SD10 + NI-BL4) and LD14 (LD14 + NI-BL4). (**B**) Blue light. The experimental layout in the plant factory (**C**): for each treatment, three replications (ten plants/replication) were located alone in an opaque compartment; the 0, 10, 20, 30, and 40 refer to the blue light with intensities of either 0, 10, 20, 30, or 40 μmol·m^−2^·s^−1^ PPFD.

As shown in Figure 11A, the 300 ± 5 μmol·m^−2^·s^−1^ PPFD of white light (WL) (~400–720 nm, and peak at 450 nm) was provided by W LEDs, while the supplemental or night-interrupting light was produced by blue (B) LEDs (MEF50120 LEDs, More Electronics Co., Ltd., Changwon, Republic of Korea). At 8:00 a.m. every day, the light period and the dark period began: the control groups were given 10 h short-day conditions (SD10) or 14 h long-day conditions (LD14) without blue light (BL) (0 μmol·m^−2^·s^−1^ PPFD); the 4 h of BL with either 10, 20, 30, or 40 μmol·m^−2^·s^−1^ PPFD of intensities was used to (1) supplement the WL at the end of the SD10 (SD10 + S-BL4) and LD14 (LD14 + S-BL4) or (2) provide night interruption (NI) in the SD10 (SD10 + NI-BL4) and LD14 (LD14 + NI-BL4) (Figure 11B). Plant factory experimental layout (Figure 11C): ten plants per replication (three replications per treatment) were grouped into opaque compartments. A spectroradiometer (USB 2000 Fiber Optic Spectrometer, Ocean Optics Inc., Dunedin, FL, USA) was used to measure the light distribution at 1 nm wavelength intervals (detects wavelengths between 200 and 1000 nm), and a quantum radiation probe (FLA 623 PS, ALMEMO, Holzkirchen, Germany) was used to measure the light intensity at three points of each light treatment at the canopy level.

### 4.3. Morphological and Growth Parameter Measurements

To ensure the strawberry plants’ complete response to each light treatment, the experimental duration was extended to 45 days. Thus, the plant morphological or growth parameters, such as shoot height, fresh weight, dry weight, average number of runners and daughter plants of mother plants, runner mean length (≥2 cm), days to the first visible runner, average number of inflorescences per mother plant, and days to the first visible inflorescences, were collected after 45 days of the light treatments. The strawberries in the SD10 treatment were not included in the runner-related statistics, while the inflorescence statistics were not included in the LD14 strawberry plants. The average number of inflorescences per mother plant contains both the blooming flowers and visible flower buds at harvest.

After carefully cleaning the divided shoot samples (without runners) of the mother plants, the dry weight of the mother plants was determined by oven drying at 85 °C for five to seven days (drying oven, Venticell-222, MMM Medcenter Geräte GmbH., Munich, Germany). For subsequent physiological investigations, the samples were immediately placed in liquid nitrogen and kept in a refrigerator at −80 °C.

### 4.4. Photosynthetic Pigment Contents

The photosynthetic pigment concentrations were measured on young mature ternately compound leaves. With minor modifications, Arnon’s study was used to determine the chlorophyll contents [89]. In brief, 0.2 g of fresh plant leaves were submerged in 2 mL of the mixture medium (45% *v*/*v* ethanol, 45% *v*/*v* acetone, 10% *v*/*v* H_2_O) and incubated at 4 °C overnight. During the incubation, mild shaking was performed with a rotator (AG, FINEPCR, Seoul, Republic of Korea). Afterwards, the supernatant was transferred to the cuvette, and the absorbance was read at 645 nm, 663 nm, and 440 nm using a spectrophotometer (Libra S22, Biochrom, Cambridge, UK). The chlorophyll—chlorophyll a, chlorophyll b, and carotenoids—contents were quantified individually using the following formulae:

Chlorophyll a = [(12.72 × OD 663 − 2.59 × OD 645) × V]/sample fresh weight

Chlorophyll b = [(22.88 × OD 645 − 4.67 × OD 663) × V]/sample fresh weight

Carotenoids = [4.7 × OD 440 − 0.27 × (Chl a + Chl b)]/sample fresh weight

where “V” is the volume of the extraction mixture solution used, and the chlorophyll content is expressed as milligram per gram of fresh leaf weight.

### 4.5. Photosynthetic Characteristics

When each plant was harvested, the Li-6400 portable photosynthesis system (LI-COR Inc., Lincoln, NE, USA) was used for photosynthetic parameter measurement of the mature, fully expended ternately compound leaves. All the parameters, including the net photosynthetic rate (*P*n), transpiration rate (*T*r), stomatal conductance (*G*s), and intercellular CO_2_ concentration (*C*i), were measured under steady light intensity 300 μmol·m^−2^·s^−1^ PPFD, an environmental temperature of 20 °C, 70 ± 5% RH, and a CO_2_ concentration of 350 ± 50 PPM from 9:00 to 11:00 in the closed-type factory.

### 4.6. Starch, Soluble Sugar, and Sucrose Contents

Younger mature ternately compound leaves were harvested 45 days after the beginning of the light treatments at night (10:00 p.m.). Approximately 0.3 g of leaf samples were used for measurement of starch and soluble sugars (as the sum of glucose, fructose, and sucrose) contents and analyzed by enzymatic assay, as in Hummel et al. [90].

### 4.7. Enzyme Activities

To measure the enzyme activity, 1.5 mL of ice-cold buffer were added to a dried frozen leaf before grinding with a pre-cooled mortar and pestle containing 2 m MEDTA, 50 mM Hepes-KOH (pH 7.5), 10 mM MgCl_2_, 10 mM dithiothreitol (DTT), 1% (*w*/*v*) Triton X-100, 1% (*w*/*v*) bovine serum albumin (BSA), 5% (*w*/*v*) polyvinylpolypyrrolidone (PVPP), 10% (*w*/*v*) glycerol, and 1 mM phenylmethylsulfonyl fluoride (PMSF). For 10 min, the extract was centrifuged at 13,000× *g* at 4 °C. Using a UV spectrophotometer (Libra S22, Biochrom Ltd., Cambridge, UK), the supernatant was used immediately for an activity assay [91]. The activities of sucrose synthase (SS) and sucrose phosphate synthase (SPS) were determined in a 1 mL reaction mixture containing 500 μL enzyme extract at 34 °C for 1 h. A 300 μL 30% (*v*/*v*) KOH was added to this mixture and was then placed in a water bath at 100 °C for 10 min, after which it was gradually cooled to room temperature. The mixture was subjected to incubation at 40 °C for 20 min after 200 μL 0.15% (*v*/*v*) of an anthrone–sulfuric acid solution was applied, and the enhancement of OD 620 nm was monitored. The phosphoenolpyruvate carboxykinase (PEPC) was assayed in a 1 mL reaction mixture consisting of 50 mM Tris-HCl (pH 8.0), 5 mM MnCl_2_, 2 mM DTT, 10 mM NaHCO_3_, 0.2 mM NADH, 5-unit NAD-MDH, and 160 μL of an enzyme extract. The reaction was initiated by adding 2.5 mM phosphoenolpyruvate (PEP). The phosphoenolpyruvate phosphatase (PEPP) was determined in a 1.5 mL reaction mixture containing 100 mM imidazole-HCl (pH 7.5), 50 mM KCl, 10 mM MgCl_2_, 0.05% (*w*/*v*) BSA, 2 mM DTT, 150 μM NADH, 1 unit LDH, 2 mM ADP, and 150 μL of an enzyme extract. The reaction was initiated with 2 mM PEP, and the increase in the OD 412 nm was monitored. The description above of the enzymatic activities was conducted in accordance with the directions provided by Feng et al. [91] and Yang et al. [92]. Additionally, the adenosine diphosphate glucose pyrophosphorylase (ADPGPPase), soluble starch synthase (SSS), and uridine diphosphate glucose pyrophosphorylase (UDGPPase) activities were also measured according to Doehlert et al. [93] and Liang et al. [94].

### 4.8. Real-Time Quantitative PCR Verification

Based on manufacturer’s instructions, an RNeasy Plant Mini Kit (Takara Bio Inc., Tokyo, Japan) was used to extract the total RNA, followed by treatment with RNase-free DNase (Takara Bio Inc., Tokyo, Japan). A cDNA product of 1 μg total RNA was synthesized using PrimeScript ^®^ Reverse Transcriptase (Takara Bio Inc., Tokyo, Japan). In a Roche Light Cycler 96 real-time fluorescence quantitative PCR instrument (Roche, Basel, Switzerland), 5 μL of cDNA diluted 10-fold was used for 15 μL of quantitative RT-PCR (qRT-PCR) reactions with SYBR Premix Ex TaqTM II (Takara Bio Inc., Tokyo, Japan). The 2^−∆∆Ct^ method [95] was used to determine the relative expression levels of each gene. The data were averagely normalized against the expression of the *FaEF1* (Acttin gene-1) and *FaMSI1* (Acttin gene-2) reference genes. As shown in Table 1, the primer sequences and PCR conditions were used in the analyses.

### 4.9. Statistical Analysis

All the plants were sampled randomly in this study. Data processing, plotting, and statistical analysis were performed using Excel 2016 and DPS (DPS for Windows, 2009). With a statistical program (SAS, Statistical Analysis System, V. 9.1, Cary, NC, USA), significant differences were assessed using a one-way analysis of variance (one-way ANOVA, to evaluate whether the effect of a blue light dose as a single factor has a significant effect on the variables in different experimental treatments) and Duncan’s multiple range test at a probability of (*p*) ≤ 0.05. Student’s *t*-tests (*p*) ≤ 0.05 were used to examine the differences between the treatments. In addition, all the results were obtained after repeating the experimental procedure 12 times; they are presented as the mean ± standard error.

## 5. Conclusions

In conclusion, the S-BL or NI-BL interacts with intensity under various photoperiods, affecting the flowering and runnering of seasonal strawberry plants differently. Generally, whether S-BL or NI-BL, BL (20) was the best performer for runnering, leading to more runners in both the LD and SD conditions. For flowering, except for treatment LD14 + S-BL, BL (20) was still the key light intensity. From BL (20) to BL (40), flowering was significantly promoted, especially when BL acted as the night interruption, regardless of the photoperiod. In this study, at the harvest stage, more inflorescences and runners were observed in the LD14 + NI-BL4 treatment; the LD14 + NI-BL (20) caused the maximum number of those. Moreover, SD10 + NI-BL4 was slightly inferior to LD14 + NI-BL4 in increasing the number of inflorescences and runners, but it caused earlier flowering. Additionally, S-BL and NI-BL affected the circadian rhythm expression of flowering-related genes to different degrees in the photoperiodic treatments. In the LD conditions, the application of BL stimulated the expression of LD-specific floral activator *FaFT1* and inhibited the flowering suppressor *FaTFL1* expression, resetting the expression balance between this pair of functional opposite flowering regulators, eventually resulting in the LD flowering. For runnering, the BL in non-runnering SD conditions stimulated two key genes regulating runner formation in the GA pathway, including one *LAM* gene, *FaGRAS32*, and one GA biosynthesis gene, *FaGA20ox4*, eventually resulting in the SD runnering. In addition, the positive effects of BL on enhancing plant photosynthesis and promoting carbohydrates also provided an abundant energy supply for the flowering and runnering processes. In future studies, BL modulating the trade-off between flowering and runnering needs to be explored in-depth. In particular, the effective light intensity of BL in stimulating flowering- or runnering-related gene expression and the associated plant hormone syngenesis remains to be further explored.

## Data Availability

Data are contained within the article.
